# Prognostic value of bioelectrical impedance analysis in head and neck cancer patients undergoing radiotherapy: a VALOR® study

**DOI:** 10.3389/fnut.2024.1335052

**Published:** 2024-02-22

**Authors:** Inmaculada Prior-Sánchez, Aura Dulcinea Herrera-Martínez, María Teresa Zarco-Martín, Rocío Fernández-Jiménez, Montserrat Gonzalo-Marín, Araceli Muñoz-Garach, Francisco Javier Vilchez-López, Manuel Cayón-Blanco, Ana Villarrubia-Pozo, Concepción Muñoz-Jiménez, Felisa Pilar Zarco-Rodríguez, Juana María Rabat-Restrepo, Luis Miguel Luengo-Pérez, Hatim Boughanem, María José Martínez-Ramírez, Jose Manuel García-Almeida

**Affiliations:** ^1^Endocrinology and Nutrition Department, Jaen University Hospital, Jaen, Spain; ^2^Maimonides Institute for Biomedical Research of Cordoba (IMIBIC), Cordoba, Spain; ^3^Endocrinology and Nutrition Service, Reina Sofia University Hospital, Cordoba, Spain; ^4^Department of Endocrinology and Nutrition, San Cecilio University Hospital, Granada, Spain; ^5^Granada Biosanitary Research Institute (Ibs. Granada), Granada, Spain; ^6^Malaga Biomedical Research Institute and BIONAND Platform, Endocrinology and Nutrition Department, Hospital Virgen de la Victoria de Malaga, Malaga, Spain; ^7^Department of Endocrinology and Nutrition, Quironsalud Malaga Hospital, Malaga, Spain; ^8^Department of Medicine and Dermatology, Malaga University, Malaga, Spain; ^9^Endocrinology and Nutrition Department, Malaga Regional University Hospital, Malaga, Spain; ^10^Malaga Biomedical Research Institute and BIONAND Platform, Malaga, Spain; ^11^Department of Endocrinology and Nutrition, Virgen de las Nieves University Hospital, Granada, Spain; ^12^Network Biomedical Research Center Physiopathology of Obesity and Nutrition (CiberOBN), Carlos III Health Institute, Madrid, Spain; ^13^Endocrinology and Nutrition Department, Hospital Universitario Puerta del Mar, Cadiz, Spain; ^14^Biomedical Research and Innovation Institute of Cadiz, Cadiz, Spain; ^15^Endocrinology and Nutrition Department, Hospital Universitario de Jerez de la Frontera, Cadiz, Spain; ^16^Department of Endocrinology and Nutrition, Seville Institute of Biomedicine (IBIS), Virgen del Rocio University Hospital, Seville, Spain; ^17^Department of Endocrinology and Nutrition, Valme University Hospital, Seville, Spain; ^18^Department of Endocrinology and Nutrition, Macarena University Hospital, Seville, Spain; ^19^Department of Endocrinology and Nutrition, Badajoz University Hospital, Seville, Spain; ^20^Department of Biomedical Sciences, Universidad de Extremadura, Badajoz, Spain; ^21^Spanish Biomedical Research Center in Physiopathology of Obesity and Nutrition (CIBERObn), Carlos III Health Institute, Madrid, Spain; ^22^Unidad de Gestión Clinica Medicina Interna, Lipids and Atherosclerosis Unit, Maimonides Institute for Biomedical Research in Córdoba (IMIBIC), Reina Sofia University Hospital, University of Córdoba, Cordoba, Spain

**Keywords:** bioelectrical impedance analysis (BIA), nutritional assessment, phase angle (PA), body mass cell (BCM), head and neck cancer (HNC)

## Abstract

**Introduction:**

Bioelectrical impedance analysis (BIA) serves as a method to estimate body composition. Parameters such as phase angle (PA), standardized phase angle (SPA), body mass cell (BCM), BCM index (BCMI), and fat-free mass (FFM) might significantly impact the prognosis of head and neck cancer (HNC) patients. The present study aimed to investigate whether bioelectrical parameters can be used to predict survival in the HNC population and establish the optimal cutoff points for predictive accuracy.

**Methods:**

A multicenter observational study was performed across 12 tertiary hospitals in Andalusia (a region from the south of Spain). A total of 494 patients diagnosed with HNC between 2020 and 2022 at different stages were included in this study, with a minimum follow-up period of 12 months. The BIA assessment was carried out during the first 2 weeks of radical radiotherapy treatment with chemotherapy or other systemic treatments. A multivariate logistic regression analysis of overall survival, complications, hospital admission, and palliative care and its relationship with BIA nutritional assessment was performed.

**Results:**

Significant prognostic factors identified in the multivariable analysis encompassed phase angle (PA), standardized phase angle (SPA), body cell mass (BCM), and BCM index (BCMI). Lower PA and BCM values were significantly associated with adverse clinical outcomes. A BCM threshold above 17 kg/m^2^ was the most significant predictor for predicting survival within the overall HNC population. The PA values of <5.1° in male and <4.8° in female patients showed the best predictive potential for mortality. Increased PA (as a continuous variable) demonstrated a significantly reduced risk for mortality (OR, 0.64; 95% CI, 0.43–0.94; *p* < 0.05) and a decreased likelihood of hospital admission (OR, 0.75; 95% CI, 0.52–1.07; *p* < 0.05). Higher BCM correlated with a lower risk of mortality (OR, 0.88; 95% CI, 0.80–0.96; *p* < 0.01) and a diminished probability of hospital admission (OR, 0.91; 95% CI, 0.83–0.99; *p* < 0.05).

**Conclusion:**

BIA is a crucial tool in the nutritional assessment of HNC patients. BCM and PA are the main bioelectrical parameters used to predict clinical outcomes in this population. Future studies are needed to validate BIA variables in a large cohort to ensure whether early intensification of nutritional treatment would improve survival.

## Background

Patients with head and neck cancer (HNC) represent a clinical, nutritional, and metabolically heterogeneous group of tumors. This population have a high prevalence of involuntary weight loss and muscle mass (MM) and a disease-related malnutrition (DRM) rate that can reach 30–55% (1). DRM is a negative prognostic factor that leads to altered body composition, decreased physical and mental function, and impaired clinical outcomes from the disease, such as greater morbidity, poorer tolerance to cancer treatments, increased health costs, and lower quality of life and life expectancy (2–6). The importance of quantifying MM and detecting DRM has initiated a new consensus for the diagnosis of malnutrition and the search for techniques that allow early detection of changes in body composition (7). Recent scientific studies revealed the need to use other tools for the diagnosis and evaluation of malnutrition in cancer patients. These tools provide a more precise diagnosis and allow categorizing and quantifying the degree of DRM and MM loss. In this context, bioelectrical impedance analysis (BIA) is a simple and an inexpensive technique, which is available in most health centers, that provides information about body composition. By passing an electric current through the body, resistance (R) and reactance (Xc) are measured. BIA data provide information regarding different body compartments, including fat mass (FM), fat-free mass (FFM), and appendicular skeletal muscle mass (ASMM), and hydration levels, encompassing total body water (TBW), extracellular water (ECW), and intracellular water (ICW). All these parameters can help with the nutritional diagnosis. BIA not only estimates body composition and MM but also proves useful for objectifying longitudinal changes in body composition parameters (8, 9). Although BIA equipment does not measure MM directly, it can be employed for the diagnosis of malnutrition, sarcopenia, and cachexia (7, 10, 11). In 2010 (10), and after a new update in 2019 (11), the European Working Group on Sarcopenia in Older People (EWGSOP) established the diagnostic criteria for sarcopenia employed in clinical practice using state-of-the-art techniques, including the BIA. However, these definitions and cutoff points have been developed based on the research conducted in older adults and have not been extensively investigated in cancer populations. The group recognizes that more studies are necessary to validate the measurements in specific subgroups, such as those observed in patients with HNC. The new vector systems also calculate the values of phase angle (PA), standardized phase angle (SPA), and body cell mass (BCM), which are closely related to all metabolically active tissues of the body, including lean muscle mass (8, 12). PA could be considered both a prognostic and a nutritional factor in critical patients (13, 14), in various tumors (15–20), and in patients with HNC (2, 21–23). However, different PA cutoff points have been proposed. On the other hand, there is a lack of data concerning the predictive role of BCM and other bioelectrical parameters determined by BIA in the HNC population.

This study aimed to explore the prognostic value of BIA parameters in a cohort of HNC patients treated with RT. The secondary objective was to set up the predictive cutoff points of nutritional bioelectrical parameters and their correlation to clinical outcomes in this population.

## Materials and methods

### Study design

This longitudinal observational study of routine clinical practice was conducted by the “VALOR: VALoracion morfofuncional en el paciente OncoRadioterapico” group at the Departments of Endocrinology and Nutrition in 12 hospitals in Andalusia, Spain [“Virgen de la Victoria” University Hospital and Malaga Regional Hospital (Málaga, Spain), Jaen University Hospital (Jaen, Spain), “San Cecilio” Hospital and “Virgen de las Nieves” University Hospital (Granada, Spain), “Puerta del Mar” University Hospital and Jerez de la Frontera University Hospital (Cádiz, Spain), “Virgen del Rocio” University Hospital, “Virgen Macarena” and “Virgen de Valme” University Hospital (Seville, Spain), “Reina Sofía” University Hospital (Córdoba, Spain), and Badajoz University Hospital (Badajoz, Spain)] between 2020 and 2022. Before beginning the study, the approval of the local ethics committee was obtained (reference code: 2381-M1-22).

### Patient selection

A total of 494 patients diagnosed with HNC at different stages were included in the study. The diagnosis was confirmed through medical records and pathological examinations, and the biopsy samples were classified by pathologists according to the histological features following the guidelines outlined in the “World Health Organization Classification of Tumors of the Digestive System” (2016) (24). All patients were included during the first 2 weeks of radical radiotherapy treatment with chemotherapy and other systemic treatments. All patients had at least a 1-year follow-up. This follow-up entailed a clinical visit at 3, 6, and 12 months during the first year. Patients were excluded from the study if they had received more than 2 weeks of radiotherapy at the time of inclusion and if they declined to undergo nutritional measurements by BIA due to reasons related to ethnicity, extensive skin lesions, extravasation of fluids through the route and local hematomas, amputation, or having a life expectancy of less than 3 months. All patients provided written informed consent.

### Demographic and anthropometric variables

Demographic and anthropometric variables including age (years), sex (male/female), BMI (kg/m2), weight (kg), and weight loss (%) were collected.

### Clinicopathological variables

TNM stage (1–4), chemotherapy (yes/no), complications (yes/no): dermatitis (yes/no), dysphagia (yes/no), mucositis (yes/no), asthenia (yes/no), unplanned admissions (yes/no; days), exitus (yes/no), and persistence/free disease were included. The Eastern Cooperative Oncology Group (ECOG) scale was used to evaluate the functional status and quality of life (0: asymptomatic, normal activity, 1: symptomatic, may wander, 2: bedridden <50% of the day, with minimal assistance, 3: bedridden >50% of the day, with notable assistance, 4: bedridden all day, severely limited, and 5: deceased).

### Biochemical variables

Bimolecular markers such as albumin (g/ dL), prealbumin (mg/dL), proteins (g/dL), total cholesterol (mg/dL), creatinine (mg/dL), glomerular filtration rate (ml/min), urea (mg/dL), glucose (mg/dL), C-reactive protein (CRP, mg/dL), TSH (μUI/mL), and HbA1c (%) were measured.

### Body composition measurements

Body weight was determined using the BIA device’s weight scale measured to the nearest 0.1 kg, with patients standing in the center of the platform without shoes and wearing only underwear. Height was measured using a seca stadiometer (Hamburg, Germany). Abdominal circumference was measured while patients lay on their backs at the level of the belly button. Body composition analysis was performed using a 50-kHz phase-sensitive impedance analyzer (BIA 101 Whole Body Bioimpedance Vector Analyzer, AKERN, Florence, Italy) that delivers 800 μA using tetrapolar electrodes positioned on the right hand and right foot. The values of phase angle (PA,°), standardized phase angle (SPA), body cell mass (BCM, kg), body cell mass index (BCMI, kg/m^2^), fat mass (FM, kg), fat mass index (FMI, kg/m^2^), fat-free mass index (FFMI, kg/m^2^), appendicular skeletal muscle mass (ASMM, kg), skeletal muscle index (SMI, kg/m^2^), total body water (TBW, kg), extracellular water (ECW, kg), intracellular water (ICW, kg), hydration levels (%), reactance (Xc, Ω/m), and resistance (Rz, Ω/m) were obtained. All BIA measurements were obtained with patients in a supine position on a hospital bed. To stabilize BIA values [±2 Ω for resistance (Rz) and ± 1 Ω for Xc (reactance)], patients remained in a supine position for 5 min before the BIA measurements were obtained, as fluid shifts occur after moving from standing to recumbence and directly affect the R and Z values. The BIA measurements of patients were standardized for sex and age using the data collected from healthy Italian adults (25). PA is expressed in degrees as arctan (Xc/Rz) × (180o/π). A standardized PA value (SPA) for each individual was determined from the sex- and age-matched reference population value by subtracting the reference PA value from the observed patient PA value and dividing the result by the respective age- and sex-reference standard deviation (SD). The technical accuracy of the BIA instrument was daily assessed using a precision circuit supplied by the BIA device manufacturer (AKERN, Florence, Italy). All measured Rz and Xc values were consistently ±1 Ω of the 385 Ohm reference value. *In vivo* reproducibility of the BIA measurements was also determined, with coefficients of variation (CV) of 1–2% for Rz and Xc.

Muscle ultrasonography of the quadriceps rectus femoris (QRF) of the lower extremity was performed using a 10–12 MHz probe and a multifrequency linear matrix (Mindray Z60, Madrid, Spain) in all subjects, who were positioned in a supine position. The evaluation was performed without compression at the level of the lower third from the superior pole of the patella and the anterior superior iliac spine, measuring the anteroposterior muscle thickness, circumference, and cross-sectional area (26). The ultrasonography was performed by a specific physician who was trained in this technique previously. The probe was aligned perpendicular to the longitudinal and transverse axes in the QRF, such as rectus femoris cross-sectional area (RF-CSA), rectus femoris circumference (RF-CIR), RF-axis (X-axis and Y-axis), and L-SAT (subcutaneous fat of the leg). For each parameter, three measurements were performed, and the mean was calculated. For the evaluation of the adipose tissue in the abdominal area, we measured the midpoint between the xiphoid appendix and the navel to capture the image, where total subcutaneous abdominal fat (T-SAT), superficial subcutaneous abdominal fat (S-SAT), and preperitoneal or visceral fat (VAT) data will be measured in centimeters (24). To calculate the global adipose tissue (GAT) and GAT index (GATi), the T-SAT, L-SAT, and VAT were added together, while for GATi, the sum of T-SAT, L-SAT, and VAT was divided by the individual’s height.

### Functional assessment

Handgrip strength (HGS) was measured using a Jamar hand dynamometer (Asimow Engineering Co., Los Angeles, CA, USA). Grip strength was calculated in a seated position with the elbow flexed at 90° in the dominant hand. Patients were instructed to perform three maximal isometric contractions with brief pauses between measurements, and the maximum and mean values were recorded. The Timed Up and Go test was selected to evaluate functional capacity. It was performed with patients seated in a chair. The time taken to get up, walk 3 m, turn around, walk another 3 m, and sit back down was measured in seconds.

### Assessment of nutritional status and definition of clinical outcomes

To diagnose malnutrition based on the GLIM criteria (7), weight loss, reduced BMI, and reduced muscle mass were categorized as phenotypic criteria, while reduced food intake/ assimilation and disease burden/inflammation were classified as etiologic criteria. For the diagnosis of malnutrition, the combination of at least one phenotypic criterion and one etiological criterion was required. To detect the reduced food intake, a dietary survey was conducted, and a 24-h reminder was collected for each patient. Through an online tool developed by the University of Valladolid,^1^ the percentage of macronutrientssuch as carbohydrates (g/day), proteins (g/day), lipids (g/day), fiber (g/day), and energy requirements (Kcal/day) were estimated. A low intake was considered when it represented less than 75% of the calculated requirements. Furthermore, in each clinical interview, the presence of gastrointestinal symptoms, such as dysphagia, nausea, vomiting, diarrhea, constipation, and abdominal pain, was investigated. These symptoms have been incorporated as supportive indicators into the GLIM consensus criterion to help identify poor food intake or assimilation. To detect inflammation, supportive proxy measures of inflammation (laboratory indicators) such as serum C-reactive protein (CRP), albumin, or prealbumin were also collected. The following diagnostic criteria for moderate and severe cases were used: Moderate cases included a BMI < 20 and age < 70 years, or FFMI <17 in male subjects with weight loss between 5 and 10%, and a BMI < 22 and age ≥ 70 years, or FFMI <15 in female subjects with weight loss between 5 and 10%. Severe cases were diagnosed with a BMI < 18.5 and age < 70 years with weight loss greater than 10%, and a BMI < 20 and age ≥ 70 years with weight loss greater than 10%. The European Working Group on Sarcopenia in Older People’s diagnostic criteria for sarcopenia were used to assess decreased muscle strength, with a cutoff of <27 kg in male patients and < 16 kg in female patients (10). The main clinical outcomes included a mortality rate at 600 days, complications derived by the RT treatment (dermatitis, dysphagia, mucositis, and asthenia), need for hospital admission during or after treatment (yes/no), or requirement of palliative care (yes/no). Palliative nature was defined as a disease in a terminal stage with no possibility of cure with conventional treatments. In these cases, medical care was offered so as to improve the quality of life and/or mitigate symptoms. The final status was classified as free or persistence/progression of disease. Disease-free status was defined as a remission of the tumor after the initial therapy without evidence of lesions in the imaging tools or clinical examination. Persistence of disease was used to identify patients whose cancer remained stable after initial therapy in the imaging tests and clinical examination. Progression of disease was defined as worsening of the tumor (local or distant), as evidenced by imaging scans or clinical examination. The disease status was established by the responsible physician. The clinical outcomes were extracted from the hospital’s medical record.

### Statistical analysis

The results are presented as mean ± standard deviation (SD) for continuous variables and as numerical values (percentages) for categorical variables. Statistical tests such as the Student *t*-test or Wilcoxon test were employed based on the normality of the variables included. Pearson’s correlation coefficients among variables were calculated, and both linear and logistic regression analyses were conducted. A logistic regression analysis provided the odds ratio (OR) with 95% confidence intervals (CIs). The predictive capability of muscle mass variables was assessed using the receiver operating characteristic (ROC) curves and the area under the curve (AUC). Cox regression was used to analyze the association between mortality and body composition variables. Multivariate logistic regression of complications, hospital admission and palliative care and its relationship with nutritional assessment methods were performed. The results were adjusted by age, gender, BMI, hsCRP, and ECOG: [0 (comprising 217 patients) and a sum ranging from 1 to 5 (encompassing 239 patients)] (*p < 0.05; **p < 0.01; ***p < 0.001). A nomogram model predicting mortality risk was formulated and validated via multivariable COX regression analysis, utilizing the *rms* and foreign packages in R software. Decision tree analysis utilized the *rpart* package, and random forest analysis was conducted using the *Randomforest* package. All analyses and graphical representations were performed using R v.3.5.1 software (Integrated Development for R, RStudio, PBC, Boston, MA, USA). Statistical significance was set at a *p-value* of <0.05 or through the chi-squared test, when applicable.

## Results

### Characteristics of the patient

A total of 494 patients (386 men, 108 women) with a histologically confirmed HNC were included in the study. The median age of the patients was 63.9 years. Table 1 describes the baseline characteristics of the population. Demographic variables, BIA parameters, echography exploration, functional measurement, biochemical parameters, clinicopathological variables, and relevant clinical outcomes are shown in Table 1. Approximately 55% of the patients were in an advanced tumor stage (III:19.1% or IV:36.6%) at the time of diagnosis. Approximately half the patients had a normal performance status (ECOG 0), while 8.5% of the patients presented significant limitations in their health condition (ECOG≥2). The mean weight loss 3–6 months before diagnosis was 6.25%. A total of 54.6% received adjuvant chemotherapy. A total of 41% presented disease progression during the follow-up. Only 27% reached free disease status after initial treatment. Approximately 51.9% of the patients needed unintentional hospitalization, and in 63.3% of the patients, complications were revealed. Mortality at 600 days was confirmed to be at 24.9% (Table 1).

**Table 1:** Baseline characteristics of the population of study.

	**All** ***N = 494***
**Demographic variables**	
Age (years)	63.9 (10.1)
Sex (male/female)	386/108
BMI (kg/m^2^)	25.2 (4.53)
Weight loss (%)	6.25 (9.10)
**BIA**	
PA (°)	5.19 (0.91)
SPA	−0.54 (1.17)
BCM (kg)	25.5 (6.11)
FM (kg)	18.8 (8.07)
FFMI (kg/m^2^)	18.4 (2.47)
FMI (kg/m^2^)	6.66 (2.72)
BCMI (kg/m^2^)	9.09 (1.91)
SMI (kg/m^2^)	8.67 (1.52)
**Echography exploration**	
RF-CSA (cm^2^)	3.45 (1.30)
RF-CIR (cm)	8.68 (1.35)
RF-X-axis (cm)	3.65 (0.55)
RF-Y-axis (cm)	1.08 (0.37)
L-SAT (cm)	0.59 (0.30)
T-SAT (cm)	1.40 (0.58)
S-SAT (cm)	0.60 (0.29)
VAT (cm)	0.65 (0.74)
GAT (cm)	2.97 (3.37)
GATi (cm/m)	0.16 (0.27)
**Functional measurement**	
HGS max (kg)	32.9 (10.2)
HGS mean (kg)	31.2 (10.0)
TUG (s)	8.11 (2.75)
**Biochemical variables**	
Glucose (mg/dL)	99.5 (17.2)
Urea (mg/dL)	38.6 (24.6)
Creatinine (mg/dL)	0.82 (0.18)
Glomerular filtration rate	87.9 (8.64)
Pre-albumin	24.4 (7.55)
Total cholesterol (mg/dL)	187 (42.0)
Proteins (g/dL)	6.99 (0.59)
Albumin (g/dL)	4.00 (0.87)
CRP (mg/dL)	17.9 (31.2)
TSH (μUI/mL)	2.25 (6.94)
HbA1c (%)	5.97 (0.71)
**Clinicopathological variables**	
Cancer stage	
I	62 (13.4%)
II	145 (31.5%)
III	86 (18.7%)
IVA	39 (8.46%)
IVB	95 (20.6%)
IVC	34 (7.38%)
**ECOG**	
0	211 (48.2%)
1	190 (43.4%)
2	30 (6.85%)
3	5 (1.14%)
4	2 (0.46%)
**Chemotherapy**	
No	222 (45.4%)
Yes	267 (54.6%)
**Cancer complications**	
No	158 (36.7%)
Yes	272 (63.3%)
**Hospital admission**	
No	186 (48.1%)
Yes	201 (51.9%)
**Palliative**	
No	292 (75.6%)
Yes	94 (24.4%)
**Free-disease**	
No	281 (73.0%)
Yes	104 (27.0%)
**Progression**	
No	229 (59.0%)
Yes	159 (41.0%)
**Mortality**	
No	301 (75.1%)
Yes	100 (24.9%)

Data are expressed as mean ± standard deviations or percentage. Complications included dermatitis, dysphagia, mucositis, and asthenia. BCM, Body cell mass; BCMI, BCM index; BMI, body mass index; BIA, bioelectrical impedance analysis; CRP, C-reactive protein; FM, fat mass; FMI, FM index; FFMI, fat-free mass index; GAT, global adipose tissue; GATi, GAT index; HGS, hand grip strength; HbA1c, glycosylated hemoglobin; PA, phase angle; RF-CIR, circumference of quadriceps rectus femoris; RF-CSA, rectus femoris cross-sectional area; SAT, subcutaneous adipose fat of leg (L), superficial (S) and total (T) abdominal; SMI, skeletal muscle index; SPA, standardized phase angle; TSH, thyroid-stimulating hormone; TUG, Timed up and go.

### Body composition measured by BIA and principal HNC outcomes

**Figure 1:**
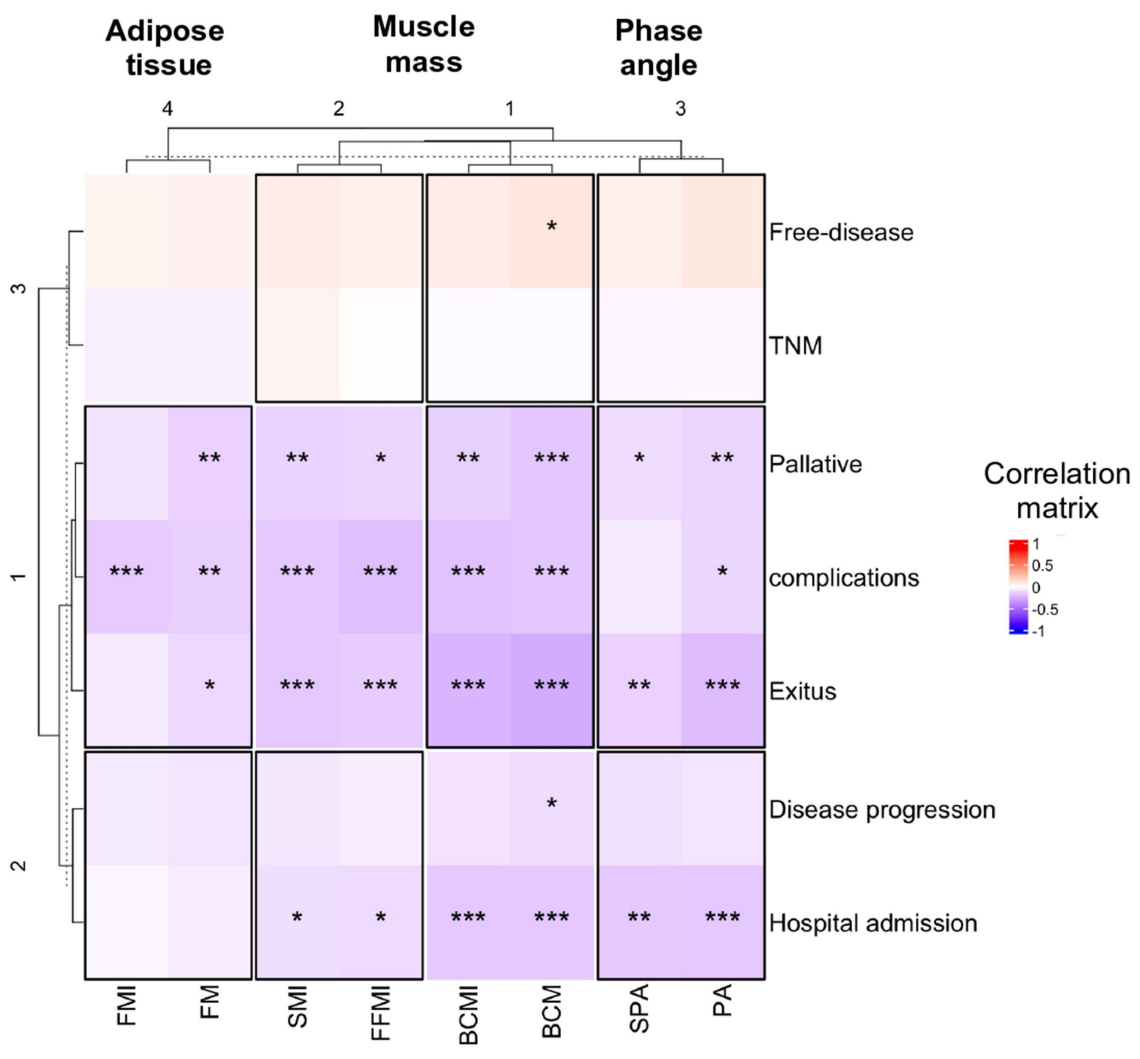
Correlation plots are presented to show the association between body composition (*X*-axis) and cancer complications related to head and neck cancer (*Y*-axis) of all participants. Pearson’s correlation coefficient was used, and an asterisk indicates a significant correlation between variables according to Pearson’s correlation test (*p < 0.05; **p < 0.01 and ***p < 0.001). BCM, body cell mass; BCMI, BCM index; FFMI, fat-free mass index; FM, fat mass; FMI, FM index; OR, odds ratio; PA, phase angle; SMI, skeletal muscle index; SPA, standardized PA.

To understand the relationship between body composition and clinical outcome, Pearson’s correlation coefficient was used. The muscle mass determined by BIA and PA showed a negative correlation with the main clinical results (complications, palliative status, hospital admission, and overall survival, see Figure 1). This negative correlation was more prominent in male participants than in female participants, as can be seen comparatively in Supplementary Figure S1 (male participants) and Supplementary Figure S2 (female participants).

The study presented the results of multivariate logistic regression analyzing the overall survival, complications, hospital admission, and palliative care in relation to BIA nutritional assessment, as detailed in Table 2. The outcomes were adjusted for age, sex, and BMI. PA, BCM, and BCMI emerged as significant prognostic factors for the overall survival. Elevated PA (as a continuous variable) exhibited a notably reduced risk for mortality (OR, 0.64; 95% CI, 0.43–0.94; *p* < 0.05), along with a decreased risk for hospital admission (OR, 0.75; 95% CI, 0.52–1.07; *p* < 0.05). High BCM was associated with a lower risk of mortality (OR, 0.88; 95% CI, 0.80–0.96; *p* < 0.01) and a reduced risk for hospital admission (OR, 0.91; 95% CI, 0.83–0.99; *p* < 0.05). On the other hand, SPA only revealed a significant low reduction for hospital admission (OR, 0.75; CI, 0.56–0.99; *p* < 0.05). Additionally, BCMI was identified as a significant prognostic factor for survival but not for other clinical outcomes. No statistically significant differences were observed among the remaining clinical variables analyzed (Table 2).

**Table 2:** Multivariate logistic regression of nutritional assessment methods and principal head and neck cancer outcomes.

	**Mortality**	**Complications**	**Hospital admission**	**Palliative care**
	OR (95% CI)	OR (95% CI)	OR (95% CI)	OR (95% CI)
Phase angle				
PA	0.64 (0.43–0.94)*	1.13 (0.78–1.64)	0.75 (0.52–1.07)*	0.88 (0.56–1.36)
SPA	1.00 (0.98–1.03)	1.05 (0.80–1.40)	0.75 (0.56–0.99)*	0.86 (0.61–1.17)
Muscle mass				
BCM	0.88 (0.80–0.96)**	1.04 (0.96–1.13)	0.91 (0.83–0.99)*	0.94 (0.85–1.04)
BCMI	0.99 (0.96–0.99)*	1.12 (0.88–1.44)	0.81 (0.62–1.04)	0.93 (0.68–1.26)
FFMI	1.00 (0.98–1.03)	1.13 (0.89–1.44)	0.96 (0.75–1.23)	1.06 (0.78–1.44)
SMI	1.00 (0.97–1.03)	1.16 (0.86–1.60)	1.01 (0.74–1.37)	1.10 (0.76–1.58)
Adipose tissue				
FM	1.00 (0.97–1.03)	0.97 (0.90–1.03)	0.97 (0.91–1.04)	0.95 (0.87–1.03)
FMI	1.01 (0.98–1.41)	0.88 (0.69–1.12)	1.03 (0.81–1.32)	0.94 (0.69–1.27)

Cox regression was used to analyze the association between mortality and body composition variables. Multivariate logistic regression of complications, hospital admission, and palliative care and its relationship with nutritional assessment methods. The results were adjusted by age, sex, BMI, hsCRP, and ECOG: [0 (comprising 217 patients) and a sum ranging from 1 to 5 (encompassing 239 patients)] (*p < 0.05; **p < 0.01; ***p < 0.001). BCM, Body cell mass; BCMI, BCM index; BMI, body mass index; FFMI, fat-free mass index; FM, fat mass; FMI, FM index; OR, odds ratio; PA, phase angle; SMI, skeletal muscle index; SPA, standardized PA.

### Decision tree and importance of BIA assessment to predict the risk for mortality

**Figure 2:**
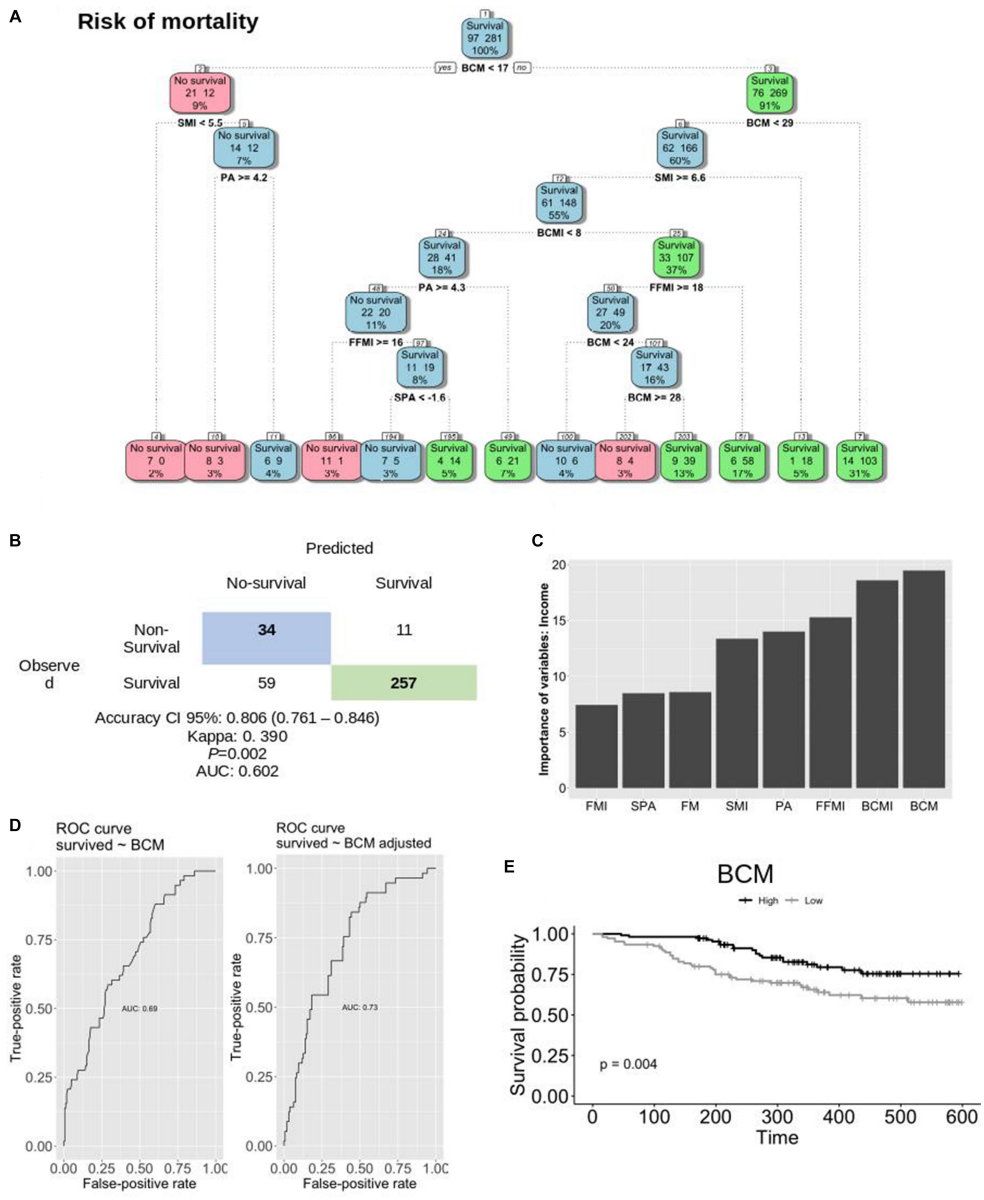
Random forest and decision tree of variables to predict mortality. **(A)** Decision tree performed with the most important variable in the model. Numbers inside the boxes indicate as follows: the left number represents the count of patients predicted as positive by the model; the right number represents the count of patients predicted as negative. The percentage indicates the proportion of individuals included in the model **(B)**. Table to calculate the accuracy of the model **(C)**. Quantitative contribution of each variable included in the analysis **(D)**. The ROC curve of BCM and adjusted BCM to predict survival **(E)**. The predictive model of BCM on survival in HNC patients. AUC, area under the curve; BCM, body cell mass; BCMI, BCM index; FFMI, fat-free mass index; FM, fat mass; FMI, FM index; OR, odds ratio; PA, phase angle; SMI, skeletal muscle index; SPA, standardized PA.

To assess the significance of BIA variables in predicting mortality, a random forest analysis was conducted (Figure 2A). The findings from this model underscore BCM as the most important predictor of survival and a determinant risk factor for mortality in HNC. Specifically, a BCM value above 17 kg correlates with a 91% chance of survival within the overall HNC population (*p* = 0.002). The accuracy of the model is shown in Figure 2B. Notably, it was confirmed that there is no evidence of multicollinearity within the model. Figure 2C illustrates the individual contribution of each variable included in the analysis. Among the variables, BCM, BCMI, FFMI, and PA demonstrate the highest discriminatory power for categorizing patients as either alive or deceased within the overall sample. Sex-based disparities are shown in Supplementary Figure S3 (male patients) and Supplementary Figure S4 (female patients). Furthermore, distinct random forest and decision tree models were developed based on sex. Figures 2D,E shows the predictive capacity of BCM on survival among HNC patients. Additionally, an ROC curve depicting the adjusted predictive ability of BCM for survival yielded an AUC of 0.73 (Figure 2D).

### Predictive value and survival outcome

**Figure 3:**
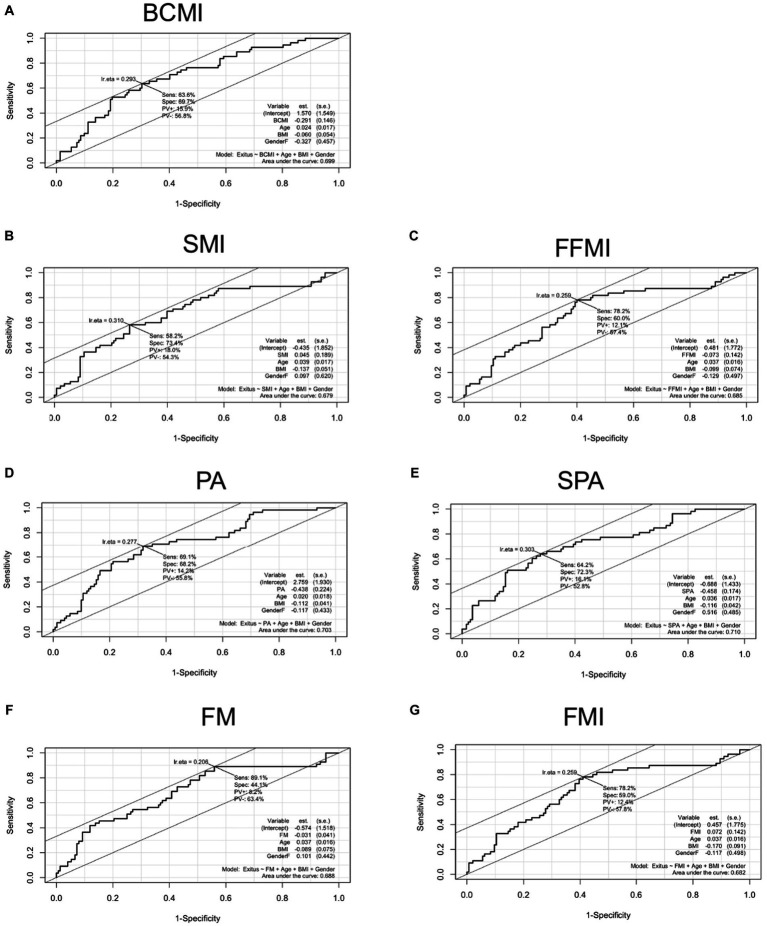
The AUC showing the predictive model of the main BIA variables, adjusted by age, sex, and BMI. **(A)** AUC body cell mass index. **(B)** AUC skeletal mass index; **(C)** AUC fat-free mass index. **(D)** AUC phase angle. **(E)**: AUC standardized phase angle. **(F)** AUC fat mass. **(G).** AUC fat mass index. AUC, area under the curve; BCM, body cell mass; BCMI, BCM index; FFMI, fat-free mass index; FM, fat mass; FMI, FM index; OR, odds ratio; PA, phase angle; SMI, skeletal muscle index; SPA, standardized PA.

The predictive value of nutritional BIA assessment on survival in HNC patients divided by sex is shown in Table 3. The cut-point PA value that gave the most accurate prediction of survival was 5.1° (sensitivity 64% and specificity 63%; *p* < 0.001). This value was lower in female patients (PA = 4.8°; *p* < 0.01) than in male patients (5.1°, *p* < 0.001), as shown in Table 3. The cutoff point for BCM was set at 28.6 kg in male patients (sensitivity 46% and specificity 83%; *p* < 0.001) and 17 kg in female patients (sensitivity 86% and specificity 57%; *p* < 0.01; Table 3). Additionally, Figure 3 shows the AUC curves of the most prognostic variables adjusted for age, sex, and BMI. Supplementary Figure S5 shows the overall survival probability at 600 days based on significant predictive variable cutoff points. The low BCMI group (≤8 kg/m^2^) exhibited a significantly lower overall survival rate compared to the normal BCMI group (>8 kg/ m^2^), with a sensitivity of 77% and a specificity of 50% (*p* < 0.001). Similarly, patients with PA >5.1° demonstrated a higher survival probability when compared to patients with PA ≤5.1°, achieving 70.3% correct predictions (*p* < 0.001; Supplementary Figure S5).

**Table 3:** Predictive value of nutritional assessment methods on survival in male and female patients with head and neck cancer.

Variables	Cutoff▴ (sens – spec)	Cutoff▴ (sens – spec)
	Males	Females
Phase angle		
PA	5.1 (0.642–0.620)***	4.8 (0.728–0.678)**
SPA	−0.75 (0.554–0.647)*	0.3 (0.589–0.785)**
Muscle mass		
BCM	28.6 (0.458–0.833)***	17.0 (0.864–0.571)**
BCMI	8.1 (0.831–0.464)***	6.7 (0.913–0.392)
FFMI	16.9 (0.861–0.380)**	18.6 (0.25–0.877)
SMI	9.0 (0.649–0.597)**	5.5 (0.744–0.983)
Muscle quality		
FM	20.3 (0.443–0.708)*	11.1 (0.909–0.296)
FMI	8.5 (0.255–0.873)	4.1 (0.41–0.269)

Receiver operating characteristic (ROC) and cutoff without adjusting for variables for the nutritional assessment methods and survival in patients with head and neck cancer. BCM, body cell mass; BCMI, BCM index; FFMI, fat-free mass index; FM, fat mass; FMI, FM index; OR, odds ratio; PA, phase angle; SMI, skeletal muscle index; SPA, standardized PA.

## Discussion

This study aimed to understand the prognostic significance of nutritional BIA assessment in patients with HNC. The findings from this study indicated that low BCM and PA were correlated with cancer outcomes, including increased risks for complications, hospital admissions, and mortality. Our data suggested that PA values below 5.1° in male patients and 4.8° in female patients served as useful reference prognostic values for Andalusian patients with HNC. Moreover, a BCM cutoff point exceeding 17 kg/m^2^ emerged as the most robust predictor for survival within the overall HNC population, which is associated with a survival probability rate exceeding 90%.

BIA, an easily accessible and non-invasive method, measures the electrical properties of a patient’s tissues and has been employed extensively for assessing body composition, encompassing measurements of FFM, FM, and TBW. In recent years, BIA has been largely utilized to evaluate PA and SPA, which are identified as prognostic factors for survival in severe diseases (13, 14), head and neck cancer (5, 23, 27–33), and other tumors (15–20, 34). However, varying values have been reported in the literature. For instance, the Swedish group led by Axelsson (33) proposed a slightly higher PA cutoff value of 5.95° for predicting 5-year survival in 128 subjects with HNC. Similar findings were provided by the German study group (32), concluding that patients with normal PA (>5.0°) exhibited notably better survival than malnourished patients (PA < 5.0°); however, the study’s limited sample size of only 42 patients could impact the robustness of conclusions.

Other studies suggested lower PA thresholds as significant markers of poorer prognosis in HNC. A study by Daniel Sat-Muñoz (21) validated that patients with PA <4.42° had reduced survival (19.8 months versus 34.4 months for those with PA >4.42°) and displayed compromised anthropometric, nutritional, and inflammatory status. Similar PA cutoff values have been reported in subsequent studies (27, 29, 35). Furthermore, the prospective randomized HEADNUT trial (29) revealed that pretherapeutic PA (4.7) served as a prognostic indicator for overall survival, while post-therapeutic PA did not influence survival probability. Additionally, a study conducted by Marie Lundberg et al. (35) demonstrated that low PA (<4.5°) correlated with prolonged hospital stays (19.1 versus 8.8 days in the normal PA group).

In our study, not only PA but also other bioelectrical nutritional parameters such as BCM and BCMI emerged as significant prognostic factors for survival. The ROC curve analysis, AUC calculations, and Kaplan–Meier curves were employed to explore their predictive value. Notably, the random forest analysis revealed BCM as the most critical variable in predicting mortality, marking a notable discovery in our study as it underscores the potential utility of BCM/BCMI as prognostic factors in HNC. Few studies have specifically focused on the association between BCM and survival in HNC patients, although correlations between body composition and prognostics have been more extensively explored in oropharyngeal cancer. For instance, a study by Bing Zhuang et al. (30) found that weight loss before RT and body composition changes during RT did not significantly impact the survival outcomes. However, patients with low appendicular skeletal muscle mass index (ASMI: male <7 kg/m^2^/female <5.7 kg/m^2^) before RT exhibited poorer overall survival. Additionally, evidence exists linking PA with body composition in the HNC population. The Polish group led by Teresa Małecka-Massalska (28) reported that PA before RT served as a useful marker for identifying HNC individuals at a high risk for unfavorable changes in body composition. Patients with low PA (4.36°) displayed over 9.3-fold higher odds of BMI reduction below 18.5 kg/m^2^ and 5.9-fold and 4.2-fold higher odds of lean mass and fat mass reduction post-therapy compared to patients with higher PA values (4.98°). Arman Arab et al. (36) conducted a systematic review and meta-analysis, including 14 studies covering 2,625 participants, to evaluate the PA’s predictive ability concerning survival in cancer patients (5 out of 14 studies involved HNC patients). Their analysis confirmed PA as a significant prognostic tool for predicting oncologic patients’ survival, although PA cutoff points varied from 3.8 to 5.9. Another recent systematic review (18) assessed SPA’s role in nutritional status and clinical outcomes in cancer patients. It concluded that, while there is a growing interest in SPA, definitive conclusions regarding its accuracy in predicting clinical outcomes (including complications and survival rates) cannot be drawn due to the absence of standardized SPA cutoff values. In our report, SPA emerged as a significant factor predicting survival, with a cutoff value of −0.75 in male patients and 0.3 in female patients.

The significance of our study lies in addressing the variability seen in PA and SPA cutoff points proposed in the existing literature. Notably, we not only presented the threshold values for the primary BIA variables but also stratified these specific cutoff points by sex. Another strength of our study involved controlling for various confounding factors through a comprehensive assessment of full body composition. Surprisingly, our findings highlighted BCM as the most robust predictor of survival among patients with HNC tumors, surpassing the predictive power of PA or SPA. BCM represents the metabolically active cell mass, encompassing FFM and immune function, and signifies the cell mass involved in critical physiological processes such as O_2_ consumption, CO_2_ production, and energy expenditure. Notably, it appears relatively less influenced by non-nutritional factors (8, 12).

Moreover, the multicenter nature of our study, involving a total of 12 tertiary hospitals and encompassing nearly 500 patients, reinforces the robustness of our findings. However, our study has several limitations. First, the relatively short follow-up time restricted our ability to assess the long-term prognosis. Second, inherent limitations of the BIA method, including potential interference from excess fluid and hydration status, could influence the parameters measured. Physiologically, AF values change depending on various factors such as sex (higher in male patients than in female patients), age (direct ratio relationship), BMI (direct correlation with extreme values, exhibiting an inverse correlation), and race. In our study, we adjusted the results for age, sex, BMI, ECOG, and hsCRP. Additionally, the smaller number of female participants might hinder the generalizability of the cutoff points to the female population, posing a limitation.

## Conclusion

In conclusion, low values of BCM and PA at diagnosis were significantly associated with shorter overall survival in patients with HNC. A BMC threshold exceeding 17 kg was associated with a 91% probability of overall survival. Our study also suggested that PA values of <5.1° in male patients and <4.8° in female patients were useful prognostic tools for patients with HNC. To the best of our knowledge, this study is one of the most powerful studies, with a large number of patients, to provide reference values for BIA to predict poor survival in patients with HNC. Therefore, BIA could be considered an essential tool for nutritional assessment in this population that will allow clinicians to implement the appropriate procedures to improve HNC patients’ survival. Further studies will be necessary to demonstrate whether intensification of nutritional treatment in this population would enhance clinical outcomes.

## Data availability statement

The original contributions presented in the study are included in the article/Supplementary material. Further inquiries can be directed to the corresponding authors.

## Ethics statement

The studies involving humans were approved by the Granada Biomedical Research Committee (CEI), Granada, Spain (Reference code: 2381-M1-22). The studies were conducted in accordance with the local legislation and institutional requirements. The participants provided their written informed consent to participate in this study. Written informed consent was obtained from the individual(s) for the publication of any potentially identifiable images or data included in this article.

## Author contributions

IP-S: Writing – original draft, Writing – review & editing, Investigation. ADH-M: Writing – original draft, Writing – review & editing, Investigation. MTZ-M: Writing – review & editing, Investigation. RF-J: Writing – original draft, Writing – review & editing, Data curation, Supervision. MG-M: Investigation, Writing – review & editing. AM-G: Investigation, Writing – review & editing. FJV-L: Investigation, Writing – review & editing. MC-B: Investigation, Writing – review & editing. AV-P: Investigation, Writing – review & editing. CM-J: Investigation, Writing – review & editing. FPZ-R: Investigation, Writing – review & editing. JMR-R: Investigation, Writing – review & editing. LML-P: Investigation, Writing – review & editing. HB: Writing – review & editing. MJM-R: Funding acquisition, Resources, Supervision, Writing – review & editing, Investigation. JMG-A: Funding acquisition, Investigation, Resources, Supervision, Writing – review & editing.

## Footnotes

^1^https://calcdieta.ienva.org/tu_menu.php
