# Supplementary material for: Prognostic value of bioelectrical impedance analysis in head and neck cancer patients undergoing radiotherapy: a VALOR® study
Source: Front Nutr. 2024 Feb 22;11:1335052. doi: 10.3389/fnut.2024.1335052 (PMC10921554; doi:10.3389/fnut.2024.1335052)
Supplement: Supplementary file 1 [file Data_Sheet_1.pdf]

Supplementary Figure S1. Correlation plots are presented to show association between body composition (X-axis) and cancer complications related to head and neck cancer (Y-axis) of males. Pearson's correlation was conducted and asterisk indicates significant correlation between variables according to the Pearson's correlation test (*p<0.05). **Abbreviations:** BCM: Body cell mass; BCMI: BCM index; FFMI: Fat-free mass index; FM: Fat mass; FMI: FM index; OR: Odds ratio; PA: Phase angle; SMI: skeletal muscle index; SPA: Standardized PA.

Supplementary Figure S2. Correlation plots are presented to show association between body composition (X-axis) and cancer complications related to head and neck cancer (Y-axis) of females. Pearson's correlation was conducted and asterisk indicates significant correlation between variables according to the Pearson's correlation test (*p<0.05). **Abbreviations:** BCM: Body cell mass; BCMI: BCM index; FFMI: Fat-free mass index; FM: Fat mass; FMI: FM index; OR: Odds ratio; PA: Phase angle; SMI: skeletal muscle index; SPA: Standardized PA.

Supplementary Figure S3. Random forest and Decision tree of variable to predict mortality in males. (A) Decision tree performed with the most important variable in the model. (B) Table to calculate the precision of the model. (C) Random forest analysis to predict the importance of variables, presented as gain. **Abbreviations:** AUC: area under curve;BCM: Body cell mass; BCMI: BCM index; FFMI: Fat-free mass index; FM: Fat mass; FMI: FM index; OR:Odds ratio; PA: Phase angle; SMI: skeletal muscle index; SPA: Standardized PA.

Supplementary Figure S4. Random forest and Decision tree of variable to predict mortality in females. (A) Decision tree performed with the most important variable in the model. (B) Table to calculate the precision of the model. (C) Random forest analysis to predict the importance of variables, presented as gain. **Abbreviations:** AUC: area under curve; BCM: Body cell mass; BCMI: BCM index; FFMI: Fat-free mass index; FM: Fat mass; FMI: FM index; OR: Odds ratio; PA: Phase angle; SMI: skeletal muscle index; SPA: Standardized PA.

Supplementary Figure S5. Kaplan-Meier curves for the variables included in the study. (A) Kaplan-Meier curve cell mass index. (B): Kaplan-Meier curve skeletal mass index; (C) Kaplan-Meier curve fat free mass index (D): Kaplan-Meier curve phase angle; (E): Kaplan-Meier curve phase angle; (F): Kaplan-Meier curve fat mass; (G): Kaplan-Meier curve fat mass index **Abbreviations:** BCM: Body cell mass; BCMI: BCM index; FFMI: Fat-free mass index; FM: Fat mass; FMI: FM index; OR: Odds ratio; PA: Phase angle; SMI: skeletal muscle index; SPA: Standardized PA.

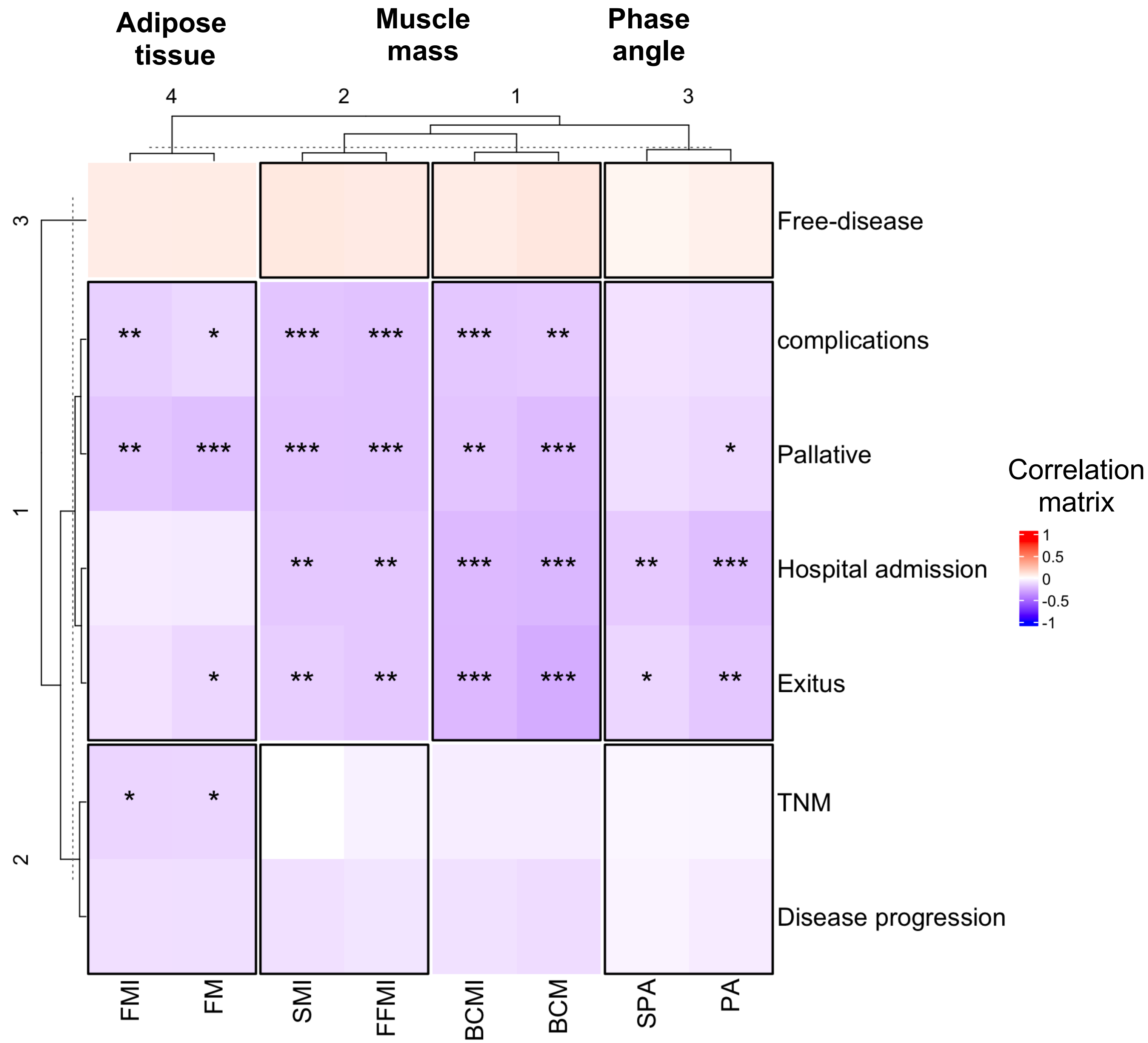

Adipose tissue
Muscle mass
Phase angle
4
2
1
3
Free-disease
complications
Pallative
Hospital admission
Exitus
TNM
Disease progression
FMI
FM
SMI
FFMI
BCMI
BCM
SPA
PA
Correlation matrix
1
0.5
0
-0.5
-1

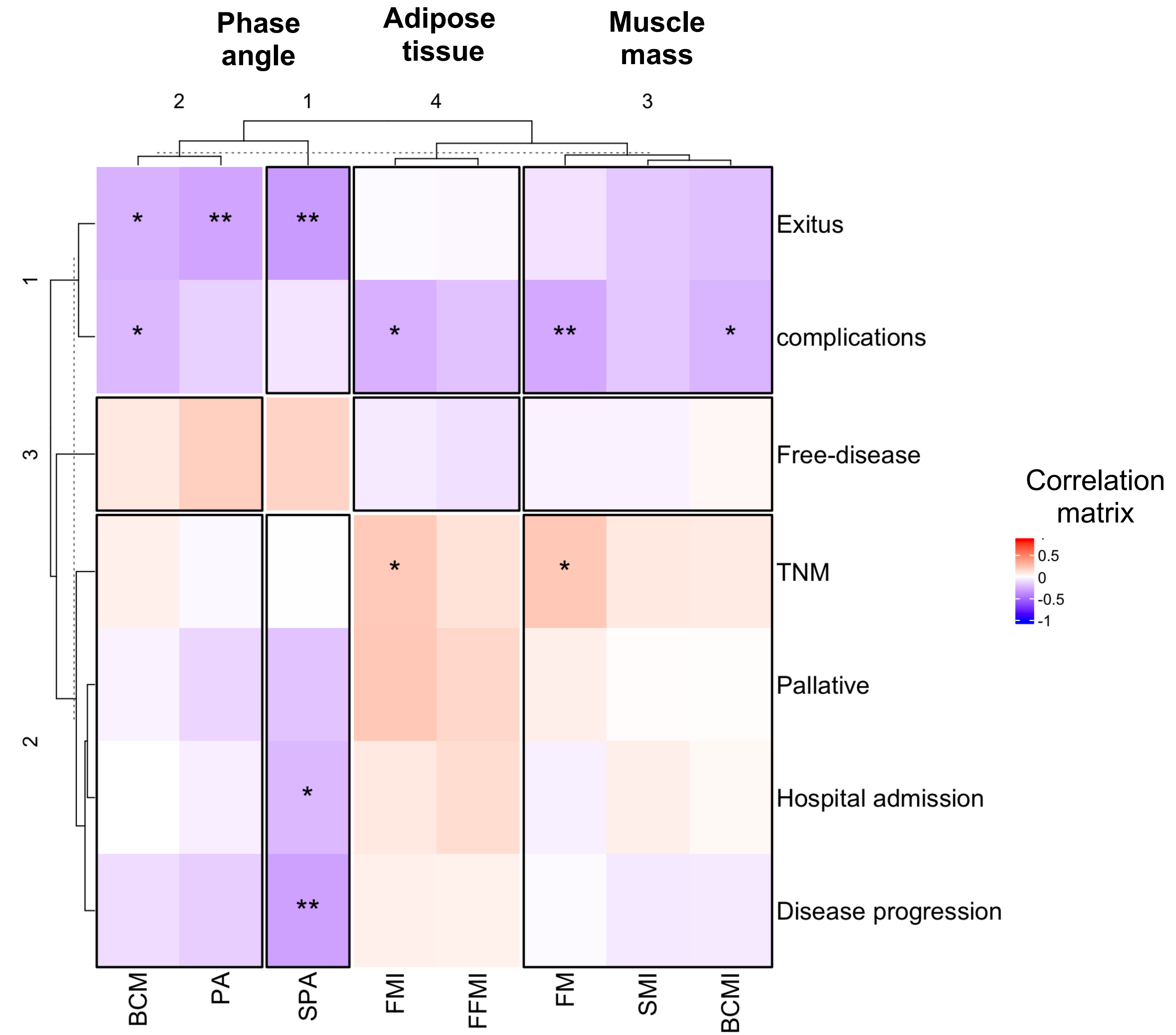
Phase angle
Adipose tissue
Muscle mass
2
1
4
3
*
**
**
*
*
**
*
*
*
*
**
Exitus
complications
Free-disease
TNM
Pallative
Hospital admission
Disease progression
BCM
PA
SPA
FMI
FFMI
FM
SMI
BCMI
Correlation matrix
0.5
0
-0.5
-1

A)

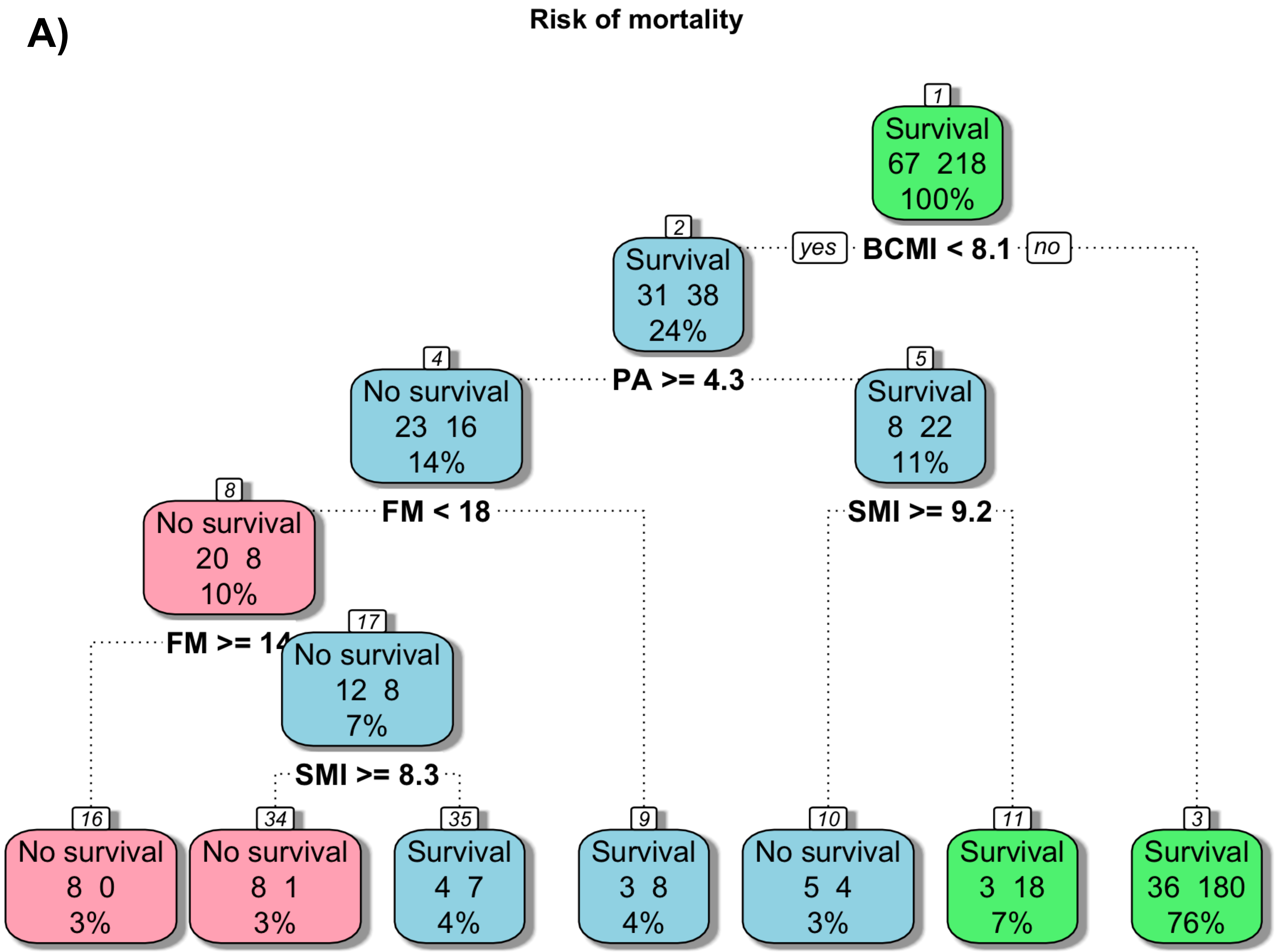

B)

		Predicted	
		No-survival	Survival
Observed	Non-Survival	**21**	5
	Survival	46	**213**
Accuracy CI 95%: 0.821 (0.771 – 0.864)<br>Kappa: 0. 369<br>*p*=0.013<br>AUC: 0.597			

C)

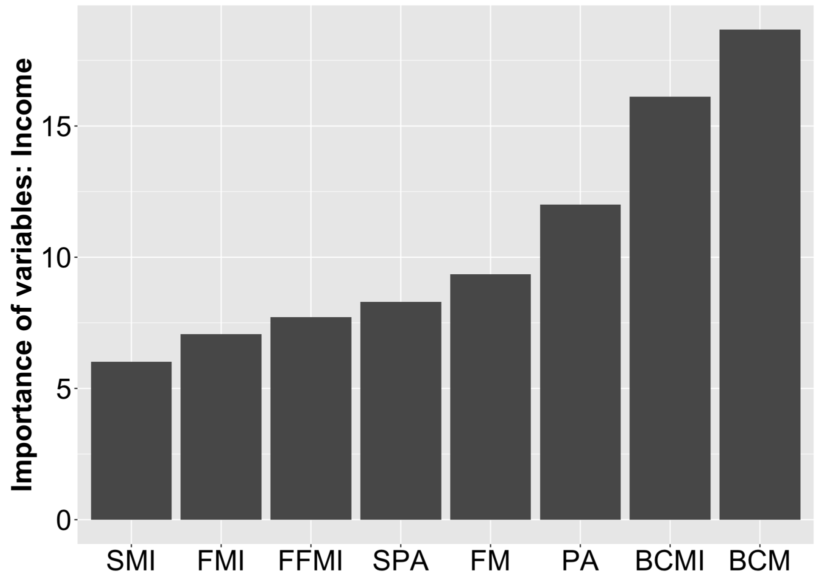

A)

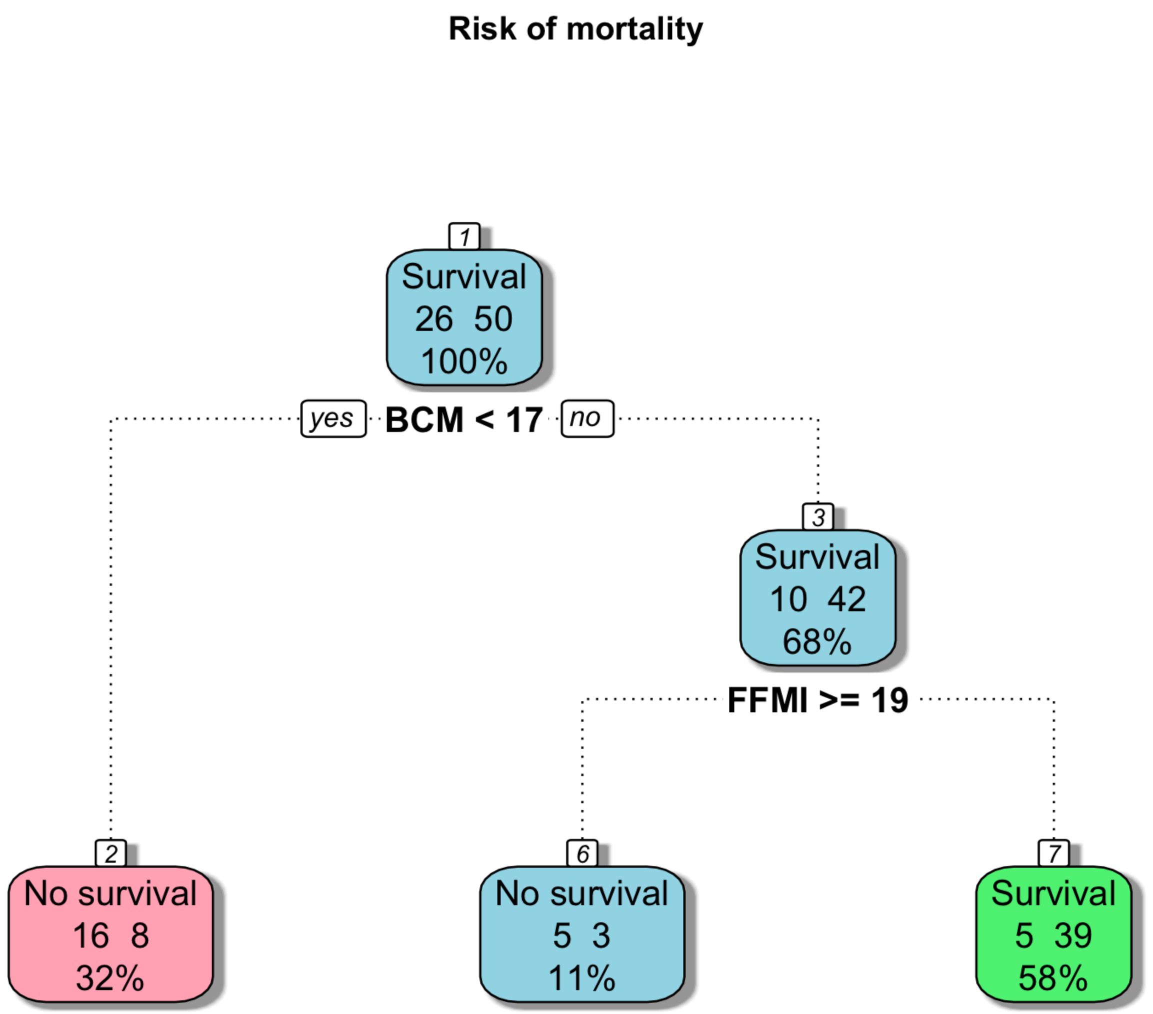

B)

		Predicted	
		No-survival	Survival
Observed	Non-Survival	**21**	11
	Survival	5	**39**
Accuracy CI 95%: 0.789 (0.681 – 0.875)<br>Kappa: 0. 557<br>*p*=0.009<br>AUC: 0.720			

C)

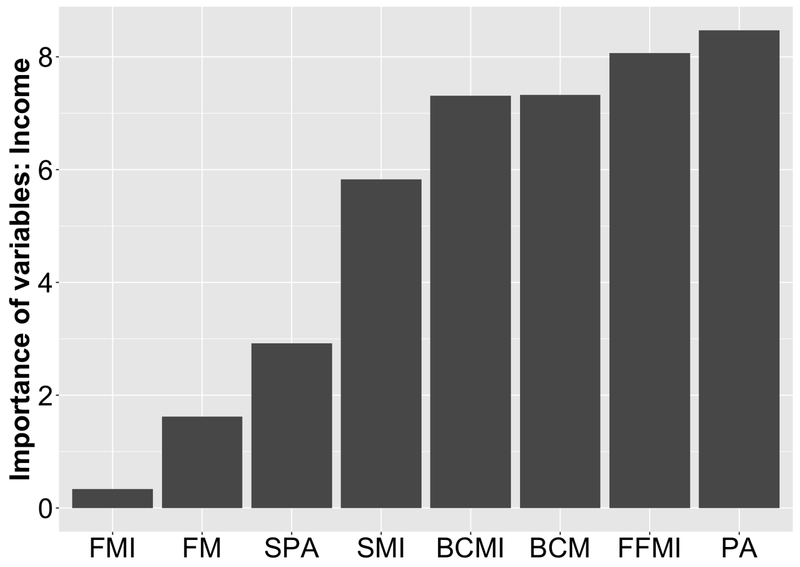

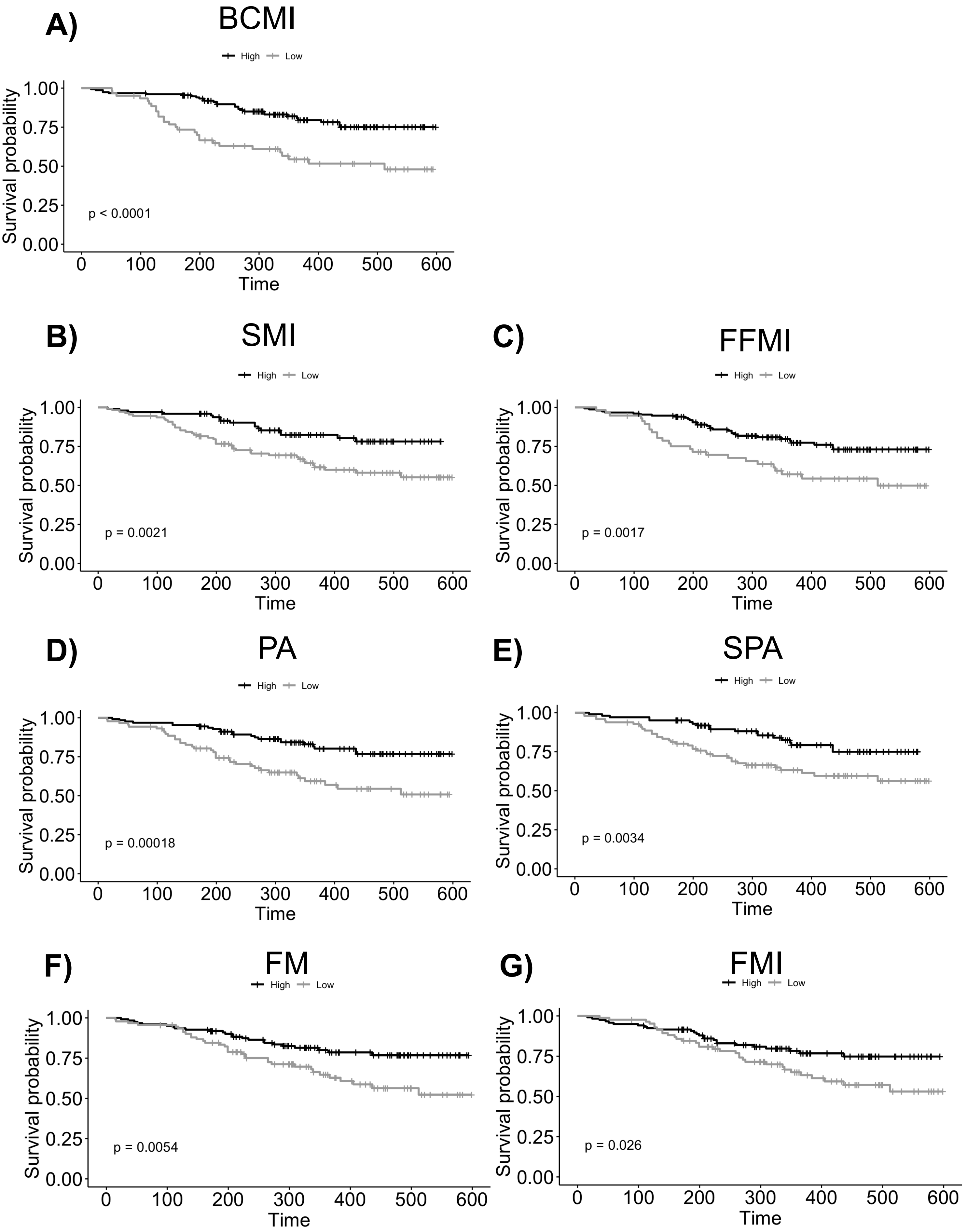
A)
BCMI
High Low
Survival probability
1.00 0.75 0.50 0.25 0.00
p < 0.0001
0 100 200 300 400 500 600
Time
B)
SMI
High Low
p = 0.0021
C)
FFMI
High Low
p = 0.0017
D)
PA
High Low
p = 0.00018
E)
SPA
High Low
p = 0.0034
F)
FM
High Low
p = 0.0054
G)
FMI
High Low
p = 0.026
